# Perspectives of ICU Patients on Deferred Consent in the Context of Post-ICU Quality of Life: A Substudy of a Randomized Clinical Trial*

**DOI:** 10.1097/CCM.0000000000006184

**Published:** 2024-01-05

**Authors:** L. Imeen van der Wal, Chloe C.A. Grim, Michael R. del Prado, David J. van Westerloo, Marcus J. Schultz, Hendrik J.F. Helmerhorst, Martine C. de Vries, Evert de Jonge

**Affiliations:** 1 Department of Intensive Care, Leiden University Medical Centre, Leiden, The Netherlands.; 2 Department of Anaesthesiology, Leiden University Medical Centre, Leiden, The Netherlands.; 3 Department of Intensive Care, Amsterdam University Medical Centre, Location AMC, Amsterdam, The Netherlands.; 4 Mahidol—Oxford Tropical Medicine Research Unit (MORU), Mahidol University, Bangkok, Thailand.; 5 Nuffield Department of Medicine, University of Oxford, Oxford, United Kingdom.; 6 Department of Medical Ethics and Health Law, Leiden University Medical Centre, Leiden, The Netherlands.

**Keywords:** critical illness, deferred consent, informed consent, intensive care unit, quality of life

## Abstract

**OBJECTIVES:**

Deferred consent enables research to be conducted in the ICU when patients are unable to provide consent themselves, and there is insufficient time to obtain consent from surrogates before commencing (trial) treatment. The aim of this study was to evaluate how former ICU patients reflect on their participation in a study with deferred consent and examine whether their opinions are influenced by the quality of life (QoL) following hospital discharge.

**DESIGN:**

Survey study by questionnaire.

**SETTING:**

Eight ICUs in The Netherlands.

**PATIENTS:**

Former ICU patients who participated in the ICONIC trial, a multicenter randomized clinical trial that evaluated oxygenation targets in mechanically ventilated ICU patients.

**INTERVENTIONS:**

Participants enrolled in the ICONIC trial in one of the eight participating centers in The Netherlands received a questionnaire 6 months after randomization. The questionnaire included 12 close-ended questions on their opinion about the deferred consent procedure. QoL was measured using the EQ-5D-5L questionnaire. By calculating the EQ-5D index, patients were divided into four QoL quartiles, where Q1 reflects the lowest and Q4 is the highest.

**MEASUREMENTS AND MAIN RESULTS:**

Of 362 participants who were contacted, 197 responded (54%). More than half of the respondents (59%) were unaware of their participation in the ICONIC study. In total 61% were content with the deferred consent procedure, 1% were not content, 25% neutral, 9% did not know, and 9% answered “other.” Those with a higher QoL were more likely to be content (*p* = 0.02). In all QoL groups, the legal representative was the most often preferred individual to provide consent.

**CONCLUSIONS:**

Former ICU patients who participated in the ICONIC study often did not remember their participation but were predominantly positive regarding the use of deferred consent. Those with a higher QoL were most likely to be content.

**KEY POINTS**

**Question:** How do former ICU patients reflect on participating in a study with deferred consent, and is their opinion dependent on their quality of life (QoL)?

**Findings:** More than half of the respondents were unaware of their participation in the ICONIC study. Patients generally found deferred consent an acceptable approach and those with higher QoL were most likely to be content.

**Meaning:** Patients were predominantly positive regarding the use of deferred consent. These findings confirm that deferred consent is a suitable option for obtaining consent from ICU patients.

Informed consent is an ethical cornerstone of medical research (1, 2). In the ICU; however, patients are often unable to provide informed consent due to their critical condition (3). An alternative would be to ask a proxy or other legal representative for consent, although clinical practice often shows that a representative is either not available or overwhelmed by the situation and therefore not able to make a well-considered decision in the narrow time window of inclusion (3, 4). For such cases, deferred consent procedures have been developed in which patients can participate in medical research before obtaining informed consent under the condition that consent is sought from the subject or their legal representative as soon as circumstances allow it (5). Ethical concerns; however, have been raised due to the fact that patients cannot express their preferences in real-time, possibly impacting their autonomy.

In recent years, the use of deferred consent procedures in clinical studies has increased considerably. Some studies have demonstrated the feasibility and acceptability of deferred consent in the ICU setting (6). In a small study from Finland, 9 of 11 patients who had survived after participating in a study on therapeutic hypothermia after cardiac arrest agreed to research in emergency settings without consent of the patient or proxy (7). Nearly all ICU patients who participated in the Normoglycemia in Intensive Care Evaluation–Survival Using Glucose Algorithm Regulation (NICE-Sugar) study would have consented to study participation if asked for consent before enrollment (8).

An important factor that may influence patients’ opinion on deferred consent procedures is their overall quality of life (QoL). Although QoL can be seriously impaired after ICU stay (9), the influence of QoL on patients’ opinion of deferred consent has not been evaluated. A prior study evaluating patients perspectives on Exception from Informed Consent (EFIC) in the Progesterone for the treatment of Traumatic Brain Injury (ProTECT III) trial found that the acceptance of the use of EFIC was generally high; however, patients with unfavorable outcomes were less accepting of their EFIC inclusion compared with those with favorable outcomes (10, 11). The ICONIC study (1, 12), a multicenter randomized controlled trial in ICU patients, comparing two oxygenation targets, allowed inclusion without prior consent and provided a population to evaluate this question. The aim of this substudy was to evaluate patients’ perspectives on deferred consent and explore the influence of QoL. We hypothesized that patients with an impaired QoL after ICU are less likely to accept participating in studies without prior consent.

## MATERIALS AND METHODS

### Participants and Setting

This is a substudy of the ICONIC trial (12), an international, multicenter, randomized, parallel-group trial, in which 664 patients were enrolled between November 2018 and November 2021. In addition, 125 patients were initially enrolled with deferred consent but subsequently excluded because consent was declined by the patient or his/her representative (**Appendix 1**, http://links.lww.com/CCM/H482). The original trial was conducted in eight ICUs in The Netherlands and one ICU in Italy. Ethical approval was granted on October 25, 2018, for all centers by the Medical Ethical Committee of Leiden, The Hague and Delft (approval number: NL65236.058.18, study title: ICONIC: Arterial oxygenation targets in mechanically ventilated patients in the ICU, a randomized controlled trial). A detailed description of the ICONIC study regulations can be found in the published protocol (1). In short, adult patients with an expected mechanical ventilation time of 24 hours or more were screened and randomized within 2 hours after intubation to either the low-oxygenation group (Pao_2_ 55–80 mm Hg or Spo_2_ 91–94%) or the high-oxygenation group (Pao_2_ 110–150 mm Hg or Spo_2_ 96–100%). Due to the emergency setting of this trial, the majority of the patients were included by deferred consent. The aim was to obtain delayed informed consent as soon as possible from either the patient or the representative. If this had not been achieved within 5 days after the study’s commencement, patients were excluded from the study. In this trial, no differences in mortality or other relevant clinical endpoints were observed between both groups.

For this substudy, participants were eligible if they were proficient in Dutch and if they were enrolled in the ICONIC study in one of the Dutch ICUs by deferred consent. Patients were excluded if informed consent was obtained before randomization. The Medical Ethical Committee of Leiden The Hague and Delft reviewed and approved the study. Written informed consent was obtained from all patients or from their legal representatives. Patients were contacted for this study between May 2019 and November 2022. This study was conducted in accordance with the Declaration of Helsinki.

### Questionnaire

To assess patient perspectives and experiences on the deferred consent procedure, a questionnaire used in a previous trial was modified and translated (**Appendix 2**, http://links.lww.com/CCM/H482) (8). The questionnaire included 12 closed-end questions and three options to provide a textual response to the choice “other.” Participants were asked whether they were aware of their participation, if they provided consent themselves or if consent was provided by their legal representative, and if they would have participated if we could have asked them before the start of the study. Responses were “Yes,” “No,” or “I don’t know.” For questions regarding the most suitable substitute decision-maker, if they were content with the decision made on their behalf, whether this was similar to the decision they would have made, and whether participating in the study would help future intensive care patients, a 5-point Likert scale ranging from “Strongly agree” to “Strongly disagree” was used. Participants were given eight response options to indicate their preferred decision-maker. Additionally, to evaluate QoL the EQ-5D-5L questionnaire was used (13) (**Appendix 3**, http://links.lww.com/CCM/H482).

### Procedures

At 6 months after enrollment in the ICONIC study, a research nurse checked in the electronic patient record if the patient was still alive and reviewed whether the patient consented to participate in the current study. Upon confirmation, patients received a questionnaire on deferred consent and the EQ-5D-5L (after 6 mo) either digitally or by post, based on their preference. Reminder telephone calls were made to patients who did not respond within 2 weeks, and the questionnaire was resent if necessary. If patients still did not respond, a final reminder was sent out, either by e-mail or telephone, 3 weeks after the initial reminder. Patients who failed to respond within 9 months of enrollment were excluded from the study. The same procedure was followed after 12 months to collect the second EQ-5D-5L questionnaire. All responses were automatically or manually registered in an electronic case report form (electronic case record form [eCRF] designed with Castor EDC) (14).

### Statistical Analysis

Data were extracted from Castor EDC (14) and analyzed using R language and environment for statistical computing, version 4.0.3 (R Foundation for Statistical Computing, Vienna, Austria). We conducted a comparative analysis of the responses obtained through the questionnaires, and aimed to evaluate acceptance of the deferred consent procedure by patients, whether patients could remember who gave consent, and the process involving the substitute decision-maker. Responses were presented for the different QoL groups. Our primary focus was to evaluate whether respondents found deferred consent acceptable and whether this was influenced by QoL.

Continuous variables were presented as means and sds, or as medians and interquartile ranges (IQRs) depending on the data distribution. Differences between groups were assessed using a Mann-Whitney *U* test. Categorical variables were presented as frequencies and percentages, and differences were evaluated using a chi-square test or Fisher exact test. Statistical significance was considered to be at a *p* value of less than 0.05. The free-text comments in the option “Other” were categorized by one investigator, and checked by a co-author. Differences were resolved by consensus.

To summarize the different health states of the EQ-5D-5L questionnaire the EQ-5D index value was calculated, including the five dimensions of health included in the EQ-5D-5L questionnaire: mobility, self-care, usual activities, pain/discomfort, and anxiety/depression (15). Each individual dimension can be scored from 1 (no problems) to 5 (extreme problems). To calculate the EQ-5D index predefined weights are assigned to each answer of the individual EQ-5D dimensions. The EQ-5D index value ranges from 0 (worst health) to 1 (full health). To categorize QoL, patients were divided into QoL quartiles based on the calculated EQ-5D index, where Q1 reflects the lowest and Q4 is the highest. The EQ-5D-5L questionnaire also includes the EQ-Visual Analog Scale (VAS), allowing patients to provide a global assessment of their health status, ranging from 0 (worst imaginable health) to 100 (best imaginable health). To examine the responses to the deferred consent questionnaire in relation to QoL, we performed an ordinal or regular logistic regression, considering EQ5D-index, age, and sex as the independent factors. To create an ordinal scale, the answer options “I don’t know,” “other” and “not applicable,” were omitted from the analysis. For one question in which the different answers could not be represented as ordinal items, a chi-square test was performed.

## RESULTS

### Participants and Quality of Life

**Figure 1.**
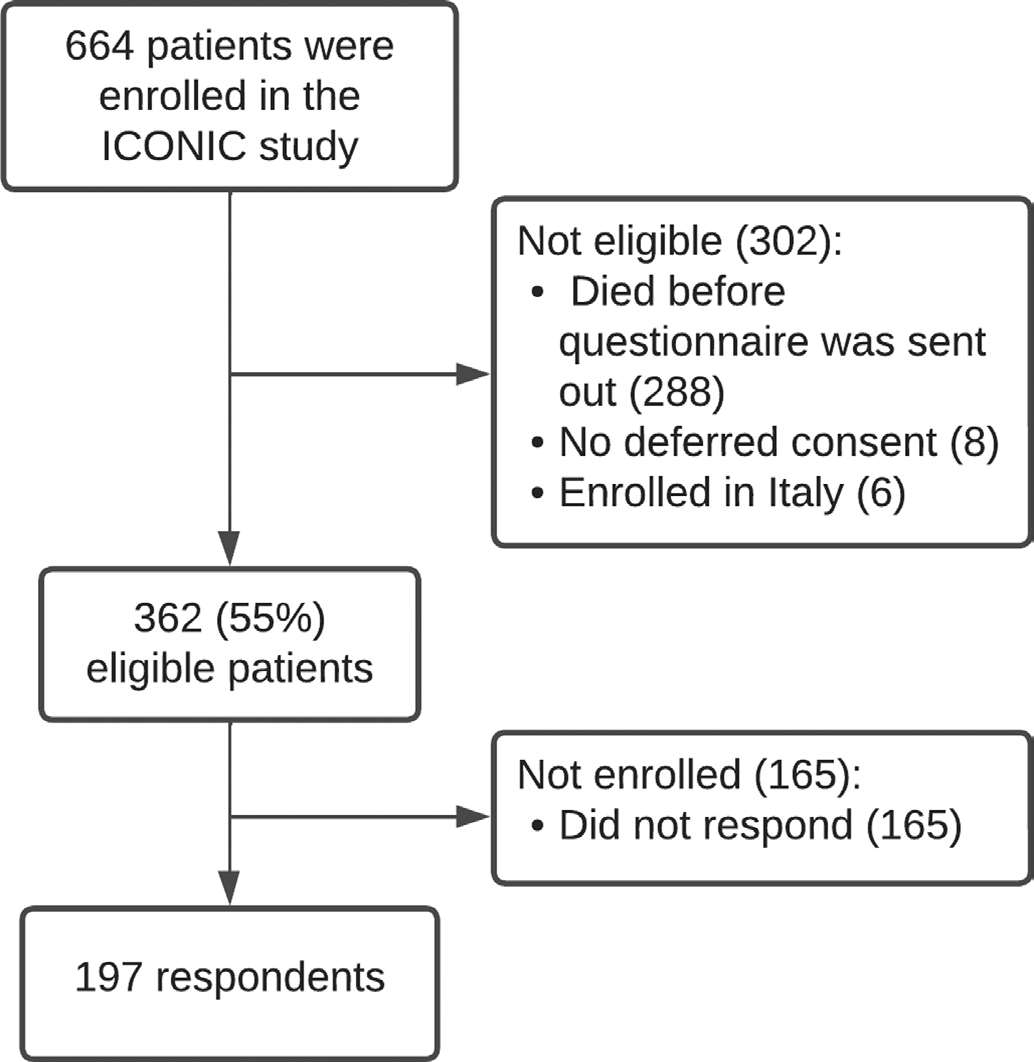
The screening and enrollment process for patients who were enrolled in the ICONIC study.

Between November 19, 2018, and November 21, 2021, a total of 664 patients were enrolled in the ICONIC study, of which 362 (55%) were eligible to participate in this substudy because deferred consent was obtained. Questionnaires were completed by 197 respondents, resulting in a response rate of approximately 54% (**Fig. 1**). The median time from enrollment until completion of the questionnaire was 29 weeks (IQR, 26–33). The median EQ-VAS score on subjective health status of all respondents was 80 (IQR, 60–90). The median EQ-5D index score of all respondents was 0.85 (IQR, 0.70–1.00). Baseline characteristics of respondents, non-responders, and the total ICONIC population are presented in **Appendix 4** (http://links.lww.com/CCM/H482). **Table 1** presents the baseline characteristics of respondents categorized by QoL group. Patients who reported a lower QoL had a longer hospital stay (*p* = 0.04). The remaining baseline characteristics were similar between groups.

**TABLE 1.** Characteristics of Respondents Per Quality of Life Group

Variable	Q1 (*n* = 49)	Q2 (*n* = 49)	Q3 (*n* = 49)	Q4 (*n* = 49)
Age (median [IQR])	63 (50–68)	62 (53–73)	63 (51–72)	67 (56–72)
Sex, female (%)	19 (39)	14 (29)	15 (31)	16 (33)
Time from randomization to informed consent (d) (median [IQR])	3 (1–5)	2 (1–4)	2 (1.75–5.25)	1 (1–4)
Apache IV score at admission (median [IQR])	75 (57–92)	77 (56–94)	73 (59–89)	77 (61–91)
Sequential Organ Failure Assessment admission score (median [IQR])	8 (6–10)	8 (6–10)	9 (7–11)	8 (7–9)
Type of admission (%)				
Medical	34 (70)	39 (80)	36 (74)	34 (69)
Emergency surgery	10 (20)	8 (16)	10 (20)	11 (22)
Elective surgery	5 (10)	2 (4)	3 (6)	4 (8)
Admission diagnosis (%)				
Sepsis	6 (12)	9 (18)	7 (14)	2 (4)
Pneumonia	9 (18)	7 (14)	9 (18)	5 (10)
Cardiac arrest	8 (16)	16 (33)	15 (31)	25 (51)
Abdominal	8 (16)	1 (2)	3 (6)	2 (4)
Neurologic	6 (12)	4 (8)	3 (6)	1 (2)
Trauma	3 (6)	3 (6)	2 (4)	1 (2)
Other	9 (18)	9 (18)	10 (20)	13 (27)
ICU length of stay (d) (median [IQR])	6.6 (4–16)	5.2 (3–12)	4.6 (3–0)	4.5 (3–8)
Hospital length of stay (d) (median [IQR])	22 (12–45)	17 (8–29)	16 (10–23)	15 (8–21)
Randomization group, high oxygenation target (110–150 mm Hg) (%)	20 (41)	27 (55)	25 (51)	25 (51)
Highest level of education completed (%)				
None	1 (2)	0 (0)	0 (0)	1 (2)
Primary school	4 (8)	5 (10)	3 (6)	4 (8)
Prevocational secondary education	13 (27)	12 (25)	9 (18)	9 (18)
Secondary vocational education	17 (35)	17 (35)	22 (45)	18 (37)
Senior general secondary education/preuniversity education	4 (8)	5 (10)	4 (8)	4 (8)
Higher professional education	5 (10)	8 (16)	5 (10)	11 (22)
University	5 (10.2)	2 (4.1)	6 (12)	2 (4)
EQ-5D-index at 6 mo (median [IQR])	0.47 (0.29–0.56)	0.79 (0.74–0.81)	0.88 (0.85–0.89)	1 (1–1)

IQR = interquartile range.s

To assess quality of life (QoL) life patients were divided into 4 QoL quartiles (Q1–Q4) based on the calculated EQ-5D-index. Q1 reflects the lowest QoL, Q4 the highest. Differences between QoL quartiles were not significant with the exception of hospital length of stay (*p* = 0.04).

### Deferred Consent Procedure

**Figure 2.**
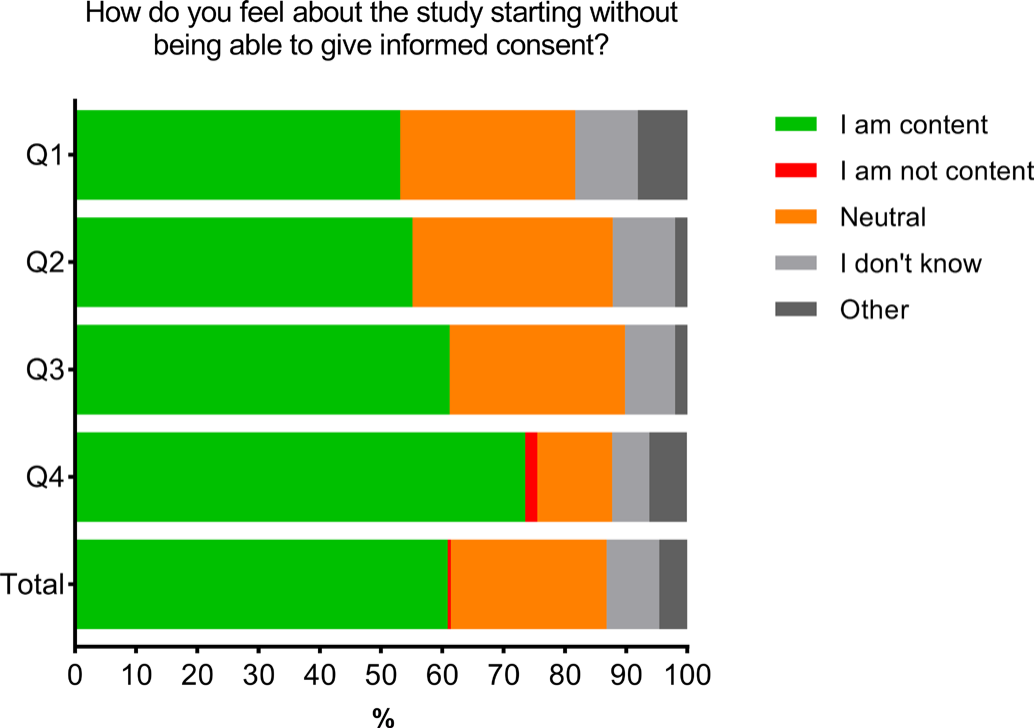
Results of the level of satisfaction regarding the deferred consent procedure. In the figure, the responses to the question: “How do you feel about the study starting without being able to give consent” are presented and stratified by quality of life (QoL) quartiles. Q1 reflects the lowest QoL, Q4 the highest.

Details of the answers to the questions in the four QoL groups are listed in **Table E2** in **Appendix 5** (http://links.lww.com/CCM/H482). Most patients were either content (61%) or neutral (25%) when asked how they felt about the ICONIC study starting without having been able to give consent (**Fig. 2**). Patients with a higher EQ-5D index were more likely to be content (*p* = 0.02). Only one person (in the highest QoL group) reported not to be content. In addition to the multiple choice answers, two respondents stated they felt forced to consent because the study had already started (**Appendix 6**, http://links.lww.com/CCM/H482). When respondents were asked if they knew they had participated in the ICONIC study, the majority of the respondents answered “No” (59%), regardless of their QoL. If consent could have been asked before start of the study, the majority of the respondents would have given consent to participate (89%). These results were similar across QoL groups. Almost all respondents either agreed (55%) or strongly agreed (35%) with the statement “Participation in the ICONIC study will help intensive care patients in the future.” These results were independent of QoL.

### Recollection of Consent

**Figure 3.**
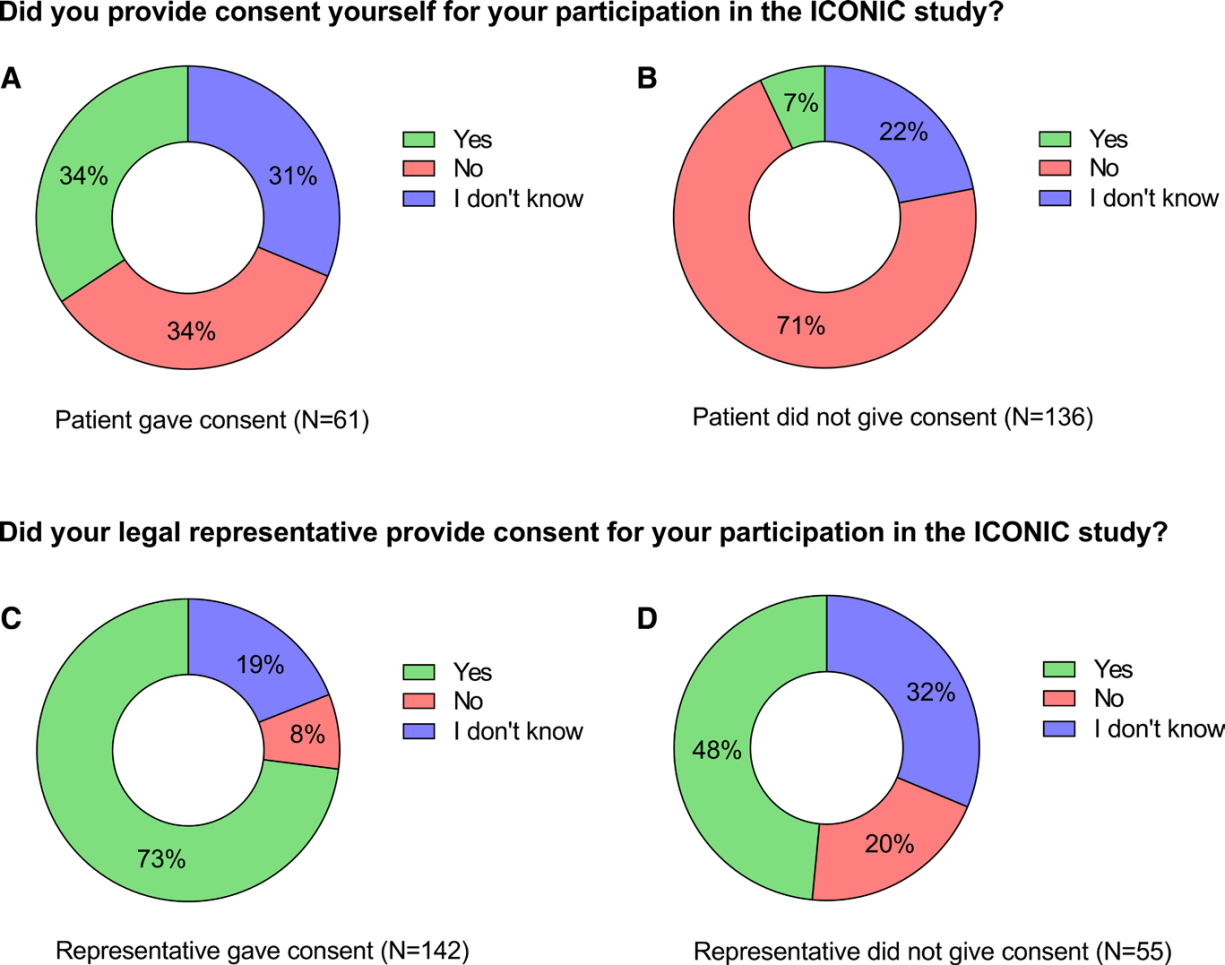
Patients’ memory of who gave consent. In the upper panel, 61 patients who had given consent (**A**) and 136 patients who had not given consent (**B**) answered the question if they had provided consent themselves to participate in the ICONIC study. The lower panel shows answers to the question if a representative had provided consent for them for 142 patients for whom a representative had given consent (**C**) and for 55 patients for whom no consent was provided by a representative (**D**).

For 197 respondents, consent was given by a representative only in 136 cases (69%), by the patient only in 55 cases (28%) and by both a representative and the patient in 6 cases (3%). More information on recollection of consent is shown in **Figure 3**. In total, 61 patients had provided written consent themselves. However, when these respondents were asked if they provided consent themselves, 21 (34%) answered “Yes,” 21 (34%) answered “No,” and 19 (31%) answered “I don’t know.” The 136 respondents who did not provide their own consent, 9 (7%) erroneously believed they did, while 97 (71%) remembered correctly, and 30 (22%) could not remember. For 142 patients, consent was provided by a legal representative. Among those 142, 104 (73%) could remember correctly (Fig. 3). In the 55 cases where a representative did not provide consent, 27 patients (48%) believed they did, 18 (32%) could not remember and 11 (20%) remembered correctly.

### Substitute Decision-Maker

Details of responses to questions about substitute decision-makers are listed in Table E2 in **Appendix 5** (http://links.lww.com/CCM/H482). In response to the question of who participants would prefer to make a decision on their behalf, the majority of respondents preferred the same legal representative who received information about their medical situation during the ICU admission (84%), irrespective of QoL. Most respondents strongly agreed (49%) or agreed (39%) that the doctors asked the right person to provide consent. Patients with a higher QoL were more likely to agree (*p* = 0.005). When asked whether the person who provided consent on their behalf made the same decision as they would have made, most respondents strongly agreed (41%) or agreed (47%). A higher QoL was associated with being more likely to agree (*p* = 0.005). The majority of the patients either strongly agreed (33%) or agreed (55%) with the decision made on their behalf, and patients with a higher QoL were more likely to agree (*p* < 0.001). Only one respondent (in QoL group Q2) disagreed.

## DISCUSSION

This study assessed the perspectives of ICU patients on the deferred consent procedure as used in the ICONIC study, and the influence of QoL on these perspectives. Despite many patients being unaware of their participation in the ICONIC study, even though deferred consent had been obtained in the process, most patients were positive regarding the use of deferred consent. Patients with a higher QoL were most likely to be content. In all QoL groups, legal representatives were the most preferred individuals to provide consent, and overall, our findings suggest general acceptance of the deferred consent procedure among ICU patients, with a trend of higher acceptance in patients with a higher QoL.

Over the years, literature has shown high levels of patient acceptance of the deferred consent procedure, with acceptance rates ranging from 82 to 95.6% (7, 8, 16–18). The level of acceptance can be influenced by several factors (6). In the Evaluation Study of Congestive Heart Failure and Pulmonary Artery Catheterization Effectiveness (ESCAPE) trial, a trial investigating endovascular thrombectomy for acute stroke patients, 78% of the patients disagreed with the use of deferred consent likely due to the high-risk nature of the intervention (19). In the Prostate Testing for Cancer and Treatment (ProTECT) trial, a trial in which EFIC was used, patients and surrogates of patients with unfavorable clinical outcomes were less accepting compared to patients with favorable outcomes (10, 11). Factors that increased the level of acceptance regarding the use of deferred consent were: perceived benefit of the research, the time-critical nature of the event, and the impact of the condition and emergency situation on the ability to provide consent (6). Other factors that were presumed to affect the level of acceptability of deferred consent were age, ethnicity, previous ICU or research experience, and gender (6). This is the first study to show that QoL affects the level of acceptability.

We hypothesized that patients with an impaired QoL after ICU were less likely to accept having participated in a study without their explicit consent. This was confirmed by the results of our study showing that patients with a higher QoL were more likely to be content with the deferred consent procedure compared with patients with a lower QoL. However, we found that it is difficult to evaluate the effect of QoL on patients’ attitudes regarding the use of deferred consent when the vast majority of the patients were content with the procedure. Furthermore, in our study, the median EQ-value and median EQ-VAS score were higher after 6 months compared with previous studies evaluating functional status and QoL after ICU stay (20, 21). Therefore, we cannot rule out that results will differ in patients with a severely impaired QoL. To add, some responses, such as “I don’t know,” “Not applicable,” and “other,” were excluded from the analysis to create an ordinal scale. Although a multinomial regression including these answers showed similar results (data not shown), it is something that needs to be considered in the interpretation of our findings.

Despite patients being mostly positive regarding the use of the deferred consent procedure, our study showed that patients were generally poor at remembering their participation, which is in line with the results of a study evaluating the deferred consent procedure in obstetric emergency research (22). It is important to note that if patients do not recall giving consent, interpreting their attitudes toward enrollment, as assessed through questionnaire responses, becomes challenging. One could argue that it is not surprising that patients do not remember participating in the study because the majority of the patients did not give consent themselves. However, also in patients who did provide consent themselves, only a third of them could remember correctly. Even more remarkable, participants were given a detailed description of the ICONIC study as part of the introduction of the questionnaire, and still they struggled to recall their participation. These findings highlight the importance of effective post-study communication methods to improve patients’ awareness of study participation. Consent is not a one-off event, but needs to be a continuous process. Therefore, in the future, we need to focus on strategies for communicating with participants after enrollment to ensure they understand what they have been part of.

Our study found that a small percentage (4.1%) of patients post hoc disagreed with study participation. These results are consistent with earlier studies indicating similar low numbers (4, 8). Even though this proportion is very low, it is important to consider when performing studies with deferred consent. In line with another study (23), a few but considerable number of respondents reported feeling pressured when asked to provide consent because the study had already started. Careful and open communication about the procedure and about the research components they can still decide about is important when they are able to give consent themselves.

The following study strengths and limitations should be considered. First, this trial is the first to integrate a QoL assessment into the evaluation of patients’ opinions of deferred consent procedures. Second, our trial had a relatively high inclusion rate compared with previous studies in this area. Additionally, earlier studies with larger sample sizes were mainly based on hypothetical scenarios with deferred consent, and did not include patients who had actual experience with the procedure. Therefore, a strength of our study is that we included critically ill patients with real-life experience with the deferred consent procedure in the ICONIC trial.

The response rate of 54% may limit the generalizability of our findings to all eligible patients who survived after participating in the ICONIC trial. Although respondents and non-responders were comparable in most baseline characteristics, it is possible that nonresponders may hold different opinions on the deferred consent procedure. Additionally, the opinions of patients who died within 6 months after inclusion or those who declined to consent were not obtained, therefore a group of patients that might have objections to deferred consent could not be included in the analysis. Also, the time from enrollment until responding to the questionnaire for the present study was 6 months and may be considered relatively long. Opinions on having participated in a study with deferred consent may change over time. We chose to study opinions at 6 months because we anticipated that administering the QoL questionnaire immediately upon or shortly after hospital discharge might result in a less accurate reflection of the actual QoL. Finally, it is important to emphasize that this analysis only included patients from The Netherlands, and it should be noted that the ICONIC trial is classified as a low-risk study. Therefore, the results from this trial may be confined to this specific cultural population and the context of a low-risk study.

## CONCLUSIONS

The present study provides more insight into the perspectives of Dutch ICU survivors on their participation in research with deferred consent. It appears that the majority of ICU patients who took part in the ICONIC trial were positive regarding the use of the deferred consent procedure, with patients with a higher QoL status 6 months post-ICU discharge being most likely to be content with the deferred consent procedure. These findings confirm that deferred consent is a suitable option for obtaining consent from ICU patients.

## ACKNOWLEDGMENTS

This study was funded by the Dutch Research Council (NWO) (project number 401.16.009). The authors want to thank E.C. Boerma, H.G. Rijnhart-de Jong, A.C. Reidinga, B.G. Loef, P.L.J. van der Heijden, M.J. Sigtermans, F. Paulus, A.D. Cornet, M. Loconte, F.J. Schoonderbeek, N.F. de Keizer, F. Bakhshi Raiez, S. Le Cessie, A. Serpa Neto, P. Pelosi, J. Wigbers, F. Termorshuizen, C. Klop, L. Dawson, E.Y. Schriel-van den Berg, E. de Vreede, J. Qualm, M. Koopmans, T. Krol, M. Rinket, J.W. Vermeijden, A. Beishuizen, J. van Holten, A.M. Tsonas, M. Botta, T. Winters, J. Horn, F. Paulus, D. Battaglini, L. Ball, and I. Brunetti from the ICONIC study group for their assistance in data collection and data verification.

## Supplementary Material
