# Supplementary material for: Perspectives of ICU Patients on Deferred Consent in the Context of Post-ICU Quality of Life: A Substudy of a Randomized Clinical Trial*
Source: Crit Care Med. 2024 Jan 5;52(5):694–703. doi: 10.1097/CCM.0000000000006184 (PMC11008447; doi:10.1097/CCM.0000000000006184)
Supplement: Supplementary file 1 [file ccm-52-0694-s001.pdf]

Table of content

Appendix 1: Flowchart ICONIC study Page 2

Appendix 2: Questionnaire deferred consent procedure ICONIC study Page 3-6

Appendix 3: EQ5D5L Questionnaire Page 7-8

Appendix 4: Baseline characteristics respondents, non-responders and total ICONIC study population. Page 9

Appendix 5. Results of the questionnaire based on Quality of life Page 10-11

Appendix 6: Verbatim responses Page 12

Appendix 1. Flowchart ICONIC study

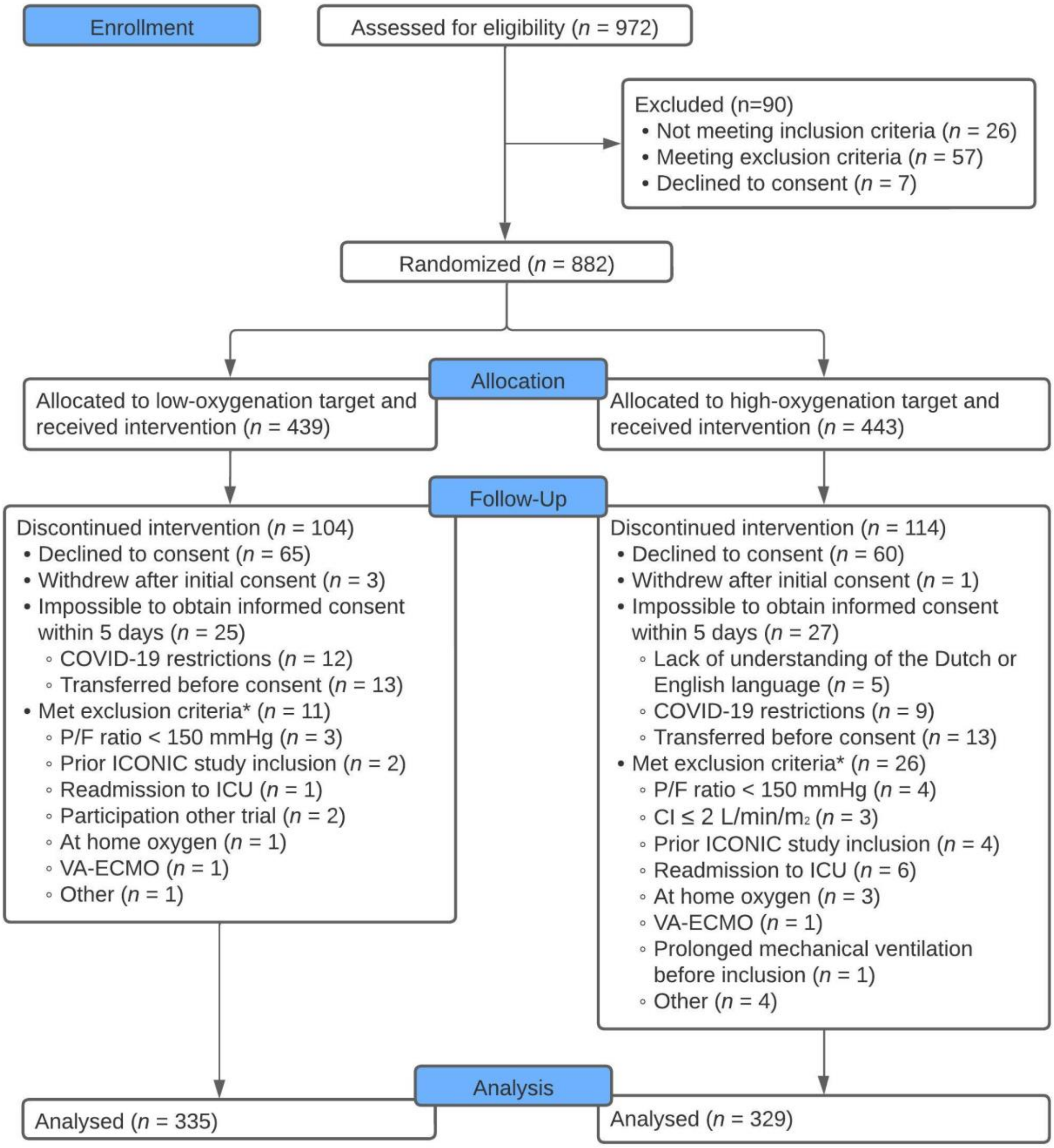

Figure E1. Consort flow diagram ICONIC study. Data was available on primary and secondary outcomes for all patients. *Patients were only withdrawn from the study if exclusion criteria were present at the time of inclusion. This was checked within 24 hours after randomization.

Appendix 2. Questionnaire deferred consent procedure ICONIC study

Introduction

About six months ago, you were admitted to the Intensive Care Unit (ICU). During this admission, you participated in a study on oxygen target levels (ICONIC study). Either you or your representative gave permission for the use of your data. Your participation in the study ended when you were discharged from the ICU. Through this survey, we aim to evaluate how you look back at participating in the study, with a specific focus on your thoughts on the consent procedure of the ICONIC study. Your answers will be treated confidentially and cannot be traced back to you personally.

What is the ICONIC study about?

ICU patients are critically ill and often require mechanical ventilation and supplemental oxygen. Research has shown that both excess and insufficient oxygen can potentially have harmful effects. However, there is a broad safe range of oxygen levels in which it remains unknown which oxygen target levels are best for patients. In the ICONIC study, two oxygen targets levels within this safe range are compared:

1. Low-normal oxygen concentration in arterial blood
2. High-normal oxygen concentration in arterial blood

Based on current scientific knowledge both targets are considered safe and are widely used in the ICU.

How is consent for research legally regulated?

Legally, obtaining the patients' consent before commencing medical scientific research is mandatory. However, in the ICU, it's often impossible to ask for consent in advance because patients are critically ill and are not in the condition to provide consent themselves. To still be able to conduct research in the ICU and improve future care, an exception is made in the law. In this case, consent is sought from a legal representative as soon as possible after the trial started, where we ask for permission to continue the trial and use the collected data for research purposes (Medical Scientific Research Act, article 6.4).

1. Did you know you participated in the ICONIC study?
   - ☐ Yes
   - ☐ No

2. Did you provide consent yourself for participation in the ICONIC study?
   - ☐ Yes
   - ☐ No
   - ☐ I don't know

3. Did your legal representative provide consent for your participation in the ICONIC study?
   - ☐ Yes
   - ☐ No
   - ☐ I don't know

4. How do you feel about the ICONIC study starting without being able to give consent?
   - ☐ I am satisfied
   - ☐ I am dissatisfied
   - ☐ Neutral
   - ☐ I don't know
   - ☐ Other, namely:

The questionnaire will continue on the next page

5. Which person would you prefer most to make the decision on your behalf to participate in the ICONIC study, given that you are unable to make the decision yourself?
   - ☐ The person who received information about my medical situation during my ICU admission (legal representative)
   - ☐ Another relative or friend
   - ☐ A Government Regulatory Authority/Guardianship Tribunal
   - ☐ The Intensive Care doctor looking after me
   - ☐ My General Practitioner
   - ☐ An independent doctor not looking after me
   - ☐ The Medical Ethics Committee
   - ☐ Someone else (please specify)

Please indicate whether you agree or disagree with the following statements

6. The doctors have asked the right person to consent on my behalf
   - ☐ Strongly agree
   - ☐ Agree
   - ☐ Neutral
   - ☐ Disagree
   - ☐ Strongly disagree
   - ☐ Not applicable, consent was only give by myself

7. My relative/friend made the same decision that I would have made, had I been able to decide.
   - ☐ Strongly agree
   - ☐ Agree
   - ☐ Neutral
   - ☐ Disagree
   - ☐ Strongly disagree
   - ☐ Not applicable, consent was only give by myself

The questionnaire will continue on the next page

8. I am content with the decision made by my relative/friend on my behalf.
   - ☐ Strongly agree
   - ☐ Agree
   - ☐ Neutral
   - ☐ Disagree
   - ☐ Strongly disagree

9. My participation in the ICONIC study will help intensive care patients in the future.
   - ☐ Strongly agree
   - ☐ Agree
   - ☐ Neutral
   - ☐ Disagree
   - ☐ Strongly disagree

10. If we could have asked you before the study started, would you have consented to participate in the ICONIC study?
    - ☐ Yes
    - ☐ No
    - ☐ I don't know

11. What is the highest educational level you attained?
    - ☐ None
    - ☐ Primary school
    - ☐ Pre-vocational secondary education
    - ☐ Secondary vocational education
    - ☐ Senior general secondary education/Pre-university education
    - ☐ Higher professional education
    - ☐ University

12. Is there anything else regarding your participation in the ICONIC study, you wanted to raise?
    - ☐ No
    - ☐ Yes, namely:

Appendix 3. EQ5D5L Questionnaire

Under each heading please tick the ONE box that best describes your health TODAY.

1. MOBILITY

☐ I have no problems in walking about
☐ I have slight problems in walking about
☐ I have moderate problems in walking about
☐ I have severe problems in walking about
☐ I am unable to walk about

2. SELF-CARE

☐ I have no problems washing or dressing myself
☐ I have slight problems washing or dressing myself
☐ I have moderate problems washing or dressing myself
☐ I have severe problems washing or dressing myself
☐ I am unable to wash or dress myself

3. USUAL ACTIVITIES (e.g. work, study, housework, family or leisure activities)

☐ I have no problems doing my usual activities
☐ I have slight problems doing my usual activities
☐ I have moderate problems doing my usual activities
☐ I have severe problems doing my usual activities
☐ I am unable to do my usual activities

4. PAIN/DISCOMFORT

☐ I have no pain or discomfort
☐ I have slight pain or discomfort
☐ I have moderate pain or discomfort
☐ I have severe pain or discomfort
☐ I have extreme pain or discomfort

5. ANXIETY/DEPRESSION

☐ I am not anxious or depressed
☐ I am slightly anxious or depressed
☐ I am moderately anxious or depressed
☐ I am severely anxious or depressed
☐ I am extremely anxious or depressed

Continue on the next page for the last question

- We would like to know how good or bad your health is **TODAY**
- This scale is numbered from 0 to 100.
- 100 means the <u>best</u> health you can imagine
  0 means the <u>worst</u> health you can imagine
- Please mark an X on the scale to indicate how your health is **TODAY**
- Now, write the number you marked in the scale in the box below

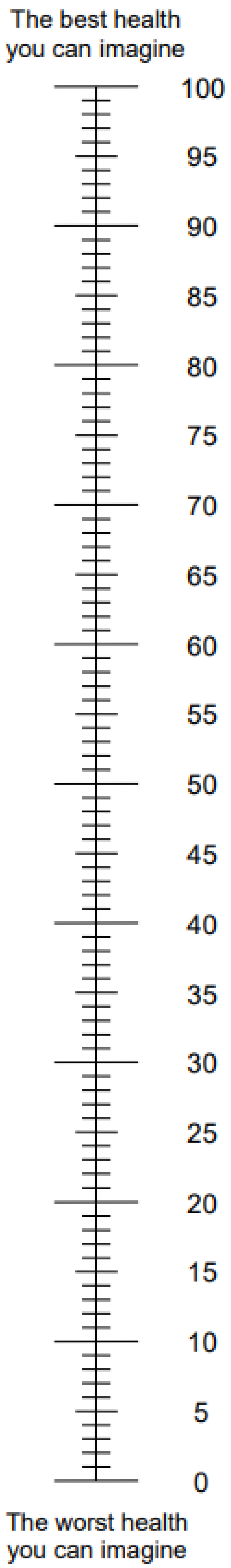

YOUR HEALTH TODAY = [ ]

Appendix 4. Baseline characteristics respondents, non-responders and total ICONIC study population.

	**Respondents (N=197)**	**Non-responders (N=165)**	**Total ICONIC study (N=664)**
Age (median [IQR])	64 [53, 71]	66 [56, 74]	67 [57, 74]
Sex = female (%)	65 (33)	54 (33)	229 (35)
Time from randomization to informed consent (days) (median [IQR])	2 [1, 5]	2 [1, 5]	2 [1, 5]
Apache IV score on admission (median [IQR])	75 [57, 92]	79 [62.8, 96]	87 [66, 109]
SOFA admission score (median [IQR])	8 [6, 10]	8 [5, 10]	9 [7, 11]
Type of admission (%)			
Medical	143 (73)	129/164 (79)	509/663 (77)
Emergency surgery	40 (20)	29/164 (18)	117/663 (18)
Elective surgery	14 (7)	6/164 (4)	37/663 (6)
Admission diagnosis (%)			
Sepsis	24 (12)	25 (15)	95 (14)
Pneumonia	30 (15)	29 (18)	97 (15)
Cardiac arrest	64 (32)	28 (17)	185 (28)
Abdominal	14 (7)	16 (10)	66 (10)
Neurologic	14 (7)	22 (13)	64 (10)
Trauma	9 (5)	8 (5)	24 (4)
Other	42 (21)	37 (22)	133 (20)
ICU length of stay (days) (median [IQR])	5.17 [2.9, 10.7]	5.9 [3.1, 13]	4.8 [2.4, 10.6]
Hospital length of stay (days) (median [IQR])	17 [9, 30]	20 [10, 33]	13 [5, 25]
Group Assignment ICONIC study (%)			
High oxygenation target (%)	97 (49)	82 (50)	329 (49)
Low oxygenation target (%)	100 (51)	83 (50)	335 (51)
Highest level of education completed (%)			
None	2/196 (1)	NA	NA
Primary school	16/196 (8)	NA	NA
Pre-vocational secondary education	43/196 (22)	NA	NA
Secondary vocational education	74/196 (38)	NA	NA
Senior general secondary education/Pre-university education	17/196 (9)	NA	NA
Higher professional education	29/196 (15)	NA	NA
University	15/196 (8)	NA	NA

Table E1. Characteristics of eligible patients who responded, eligible patients who were non-responders and the overall cohort of patients from the ICONIC study.

Appendix 5. Results of the questionnaire based on Quality of life.

Questions	Total (N=197)	Q1 (N=49)	Q2 (N=49)	Q3 (N=49)	Q4 (N=49)	P-value
**Did you know you participated in the ICONIC study?**						0.66
No	116 (59)	27 (55)	31 (63)	26 (53)	31 (63)	
Yes	81 (41)	22 (45)	18 (37)	23 (47)	18 (37)	
**Did you provide consent yourself for participation in the ICONIC study? (%)**						0.07
Yes	30 (15)	12 (25)	5 (10)	8 (16)	5 (10)	
No	118 (60)	26 (53)	30 (61)	29 (59)	33 (67)	
I don't know	49 (25)	11 (22)	14 (29)	12 (25)	11 (22)	
**Did your legal representative provide consent for your participation in the ICONIC study? (%)**						0.006
Yes	130 (66)	27 (56)	34 (70)	29 (59)	40 (82)	
No	22 (11)	9 (19)	4 (8)	7 (14)	2 (4)	
I don't know	44 (22)	12 (25)	11 (22)	13 (27)	7 (14)	
**How do you feel about the ICONIC study starting without being able to give consent? (%)**						0.02
I am content	120 (61)	26 (53)	27 (55)	30 (61)	36 (74)	
I am not content	1 (1)	0 (0)	0 (0)	0 (0)	1 (2)	
Neutral	50 (25)	14 (29)	16 (33)	14 (29)	6 (12)	
I don't know	17 (9)	5 (10)	5 (10)	4 (8)	3 (6)	
Other	9 (5)	4 (8)	1 (2)	1 (2)	3 (6)	
**Which person would you prefer most to make the decision on your behalf? (%)**						0.43*
Legal representative	165 (84)	43 (88)	41 (84)	38 (78)	43 (88)	
Other relative or friend	18 (9)	2 (4)	6 (12)	6 (12)	4 (8)	
Government regulatory/ Guardianship Tribunal	2 (1)	1 (2)	0 (0)	1 (2)	0 (0)	
Intensive Care doctor	7 (4)	2 (4)	2 (4)	3 (6)	0 (0)	
General Practitioner	1 (1)	0 (0)	0 (0)	1 (2)	0 (0)	
Independent doctor	0 (0)	0 (0)	0 (0)	0 (0)	0 (0)	
Medical ethics committee	0 (0)	0 (0)	0 (0)	0 (0)	0 (0)	
Someone else	3 (2)	1 (2)	0 (0)	0 (0)	2 (4)	
**The doctors asked the right person to consent on my behalf (%)**						0.005
Strongly agree	95 (49)	17 (35)	28 (57)	22 (45)	28 (57)	
Agree	76 (39)	22 (45)	15 (31)	19 (39)	20 (41)	
Neutral	16 (8)	4 (8)	5 (10)	6 (12)	1 (2)	
Disagree	2 (1)	2 (4)	0 (0)	0 (0)	0 (0)	
Strongly disagree	0 (0)	0 (0)	0 (0)	0 (0)	0 (0)	
NA	7 (4)	4 (8)	1 (2)	2 (4)	0 (0)	
**My relative/friend made the same decision as I would have made (%)**						0.005
Strongly agree	81 (41)	16 (33)	22 (45)	17 (35)	26 (53)	
Agree	92 (47)	25 (51)	21 (43)	26 (53)	20 (41)	
Neutral	13 (7)	3 (6)	4 (8)	4 (8)	2 (4)	
Disagree	0 (0)	0 (0)	0 (0)	0 (0)	0 (0)	
Strongly disagree	1 (1)	0 (0)	1 (2)	0 (0)	0 (0)	
NA	9 (5)	5 (10)	1 (2)	2 (4)	1 (2)	
**I am content with the decision made by my relative/friend on my behalf (%)**						<0.001
Strongly agree	64 (33)	13 (27)	14 (29)	17 (35)	20 (42)	
Agree	106 (54)	26 (53)	30 (61)	26 (53)	24 (50)	
Neutral	24 (12)	10 (20)	4 (8)	6 (12)	4 (8)	
Disagree	1 (1)	0 (0)	1 (2)	0 (0)	0 (0)	
Strongly disagree	0 (0)	0 (0)	0 (0)	0 (0)	0 (0)	
**My participation in the ICONIC study will help intensive care patients in the future (%)**						0.23
Strongly agree	68 (35)	16 (33)	15 (31)	18 (37)	19 (39)	
Agree	107 (55)	28 (57)	31 (63)	24 (49)	24 (49)	
Neutral	21 (11)	5 (10)	3 (6)	7 (14)	6 (12)	
Disagree	0 (0)	0 (0)	0 (0)	0 (0)	0 (0)	
Strongly disagree	0 (0)	0 (0)	0 (0)	0 (0)	0 (0)	
**If we could have asked you before the study started, would you have consented to participate? (%)**						0.08
Yes	174 (89)	41 (84)	47 (96)	43 (88)	43 (88)	

No	8 (4)	3 (6)	1 (2)	2 (4)	2 (4)
I don't know	14 (7)	5 (10)	1 (2)	4 (8)	4 (8)

Table E2. Influence of QoL on opinion deferred consent. QoL was divided in quartiles (Q1, Q2, Q3, Q4) based on the calculated EQ-5Dindex. Q1 reflects the lowest QoL, Q4 the highest. Textual responses for the option ‘other’ can be found in Appendix 5. P-values represent the association between QoL and given answers by an ordinal logistic regression model with QoL, age and sex as independent parameters. In order to create an ordinal or binomial scale, answers such as “I don’t know”, “Other”, and “Not Applicable” were excluded from the analysis.

*For the analysis of this question a chi-squared test was used, as no ordinal scale could be created.

Appendix 6. Verbatim responses

Verbatim responses to the question: "How do you feel about the ICONIC study starting without being able to give consent?"

<u>Group: I gave consent myself</u>

- See point 2
- Point 2

<u>Group: Not a problem</u>

- It was understood correctly, for sure
- I don't have a problem with it. However, at the time I cannot recall whether I said yes or no.
- It was weird that the study was started without me knowing (however, I do agree with my participation)
- Fine

<u>Group: Already started</u>

- They already started with the study, so my partner had to give consent

<u>Group: Mentally disabled</u>

- Mentally disabled. Does not understand what this is about.
- Patient is mentally not able to answer this question, answered by wife.

Verbatim responses to the question: "Which person would you prefer most to make the decision on your behalf to participate in the ICONIC study, given that you are unable to make the decision yourself?"

<u>Group: Wife</u>

- My wife
- My wife/partner
